# Effectiveness of precise and quantitative rapid pulmonary rehabilitation nursing program for elderly patients with lung cancer during the perioperative period: A randomized controlled trial

**DOI:** 10.12669/pjms.39.2.7103

**Published:** 2023

**Authors:** Bei Chen, Xiaoxia Yan, Xiaojun Wang, Yanjun Mao

**Affiliations:** 1 Bei Chen, Department of Thoracic Surgery, Shanghai Pulmonary Hospital, Tongji University School of Medicine, Shanghai 200433, P.R. China; 2 Xiaoxia Yan, Department of Thoracic Surgery, Shanghai Pulmonary Hospital, Tongji University School of Medicine, Shanghai 200433, P.R. China; 3 Xiaojun Wang, Department of Operating Room, Shanghai Pulmonary Hospital, Tongji University School of Medicine, Shanghai 200433, P.R. China; 4 Yanjun Mao, Department of Nursing, Shanghai Pulmonary Hospital, Tongji University School of Medicine, Shanghai 200433, P.R. China

**Keywords:** Lung Neoplasms, Nursing, Perioperative Period, Rehabilitation Nursing

## Abstract

**Objective:**

Preoperative rehabilitation should improve the functional condition of patients. Improvements in metabolism, lung mechanics, cardiovascular function, and muscle-function can be achieved by pulmonary rehabilitation. Hence, we focused on elderly patients with lung cancer undergoing surgery, and assessed the effectiveness of a rapid and precise pulmonary rehabilitation nursing program during the perioperative period.

**Methods:**

This randomized controlled trial at the department of thoracic surgery; Shanghai pulmonary hospital, China during 2021 was conducted amongst 218 elderly patients with lung cancer treated by surgical operation into either a precision quantitative nursing model nursing group (experimental group) or a perioperative routine nursing group (control group). After individual planning of the pulmonary rehabilitation nursing outpatient clinic, nurses distributed breathing trainers, instructed the patients in breathing training, and supervised the patients after the operation. For our evaluation we considered pulmonary function tests, postoperative thoracic drainage tube indwelling times, postoperative hospital stays, postoperative complication rates, and postoperative quality of life results.

**Result:**

The experimental group showed better pulmonary function, length of hospital stay, and quality of life outcomes than the control group, but the differences were not statistically significant. However, we found a significantly higher postoperative complications rate in the control group (11.9%) than in the experimental group (3.7%; p=0.02).

**Conclusion:**

Strengthening pulmonary rehabilitation nursing interventions for elderly patients with lung cancer during the perioperative period can reduce their postoperative complications and promote their rapid recovery.

***Clinical Trial:*** Registration Number - ChiCTR2100042916.]

## INTRODUCTION

Lung cancer is the most common malignancy and its morbidity and mortality rank first across several countries.^1^ Surgical removal is the most effective treatment for patients with early and mid-stage lung cancer.^1^ Elderly patients present the highest risk of having lung cancer amongst all age groups and the proportion of elderly patients with lung cancer has increased with the intensifying population aging trend.^2^ Elderly patients have a characteristic diminished lung function, multiple comorbidities and poor cognitive coordination abilities.^2^ In addition, variables such as a lung cancer that severely impairs the functional capacity, low levels of physical activity, smoking, high body mass index (obesity), and other comorbidities need to be considered for risk stratification before performing a thoracic surgery.^3^-^5^

Preoperative rehabilitation should improve a patient’s functional condition. Pulmonary rehabilitation improves the metabolism, lung mechanics, cardiovascular function, and muscle-function.^6^ Non-pharmacological interventions like exercise and functional capacity improvements before, after or during the cancer treatment or surgery are being studied.^7^

Exercise is one of the number of successful interventions improving the physical and psychological health of patients with different cancers (including lung cancer).^8^,^9^ The perioperative management of patients with lung cancer includes cardiopulmonary rehabilitation; it can reduce symptoms and minimize cancer exacerbations.^8^,^9^ Pulmonary rehabilitation can improve the exercise capacity and health-related quality of life of patients, and it reduces the presence of major symptoms like fatigue, dyspnea, depression, and others.^10^ However, the evidence for the effectiveness of specific interventions for the patients with lung cancer remain scarce.^11^ Defining the optimal perioperative exercise interventions that are feasible, acceptable and that positively promote the well-being of patients with lung cancer is important. Hence, we designed this trial to investigate the effectiveness of a precise and quantitative rapid pulmonary rehabilitation nursing program for elderly (older than 70 years) patients with lung cancer during the perioperative period.

## METHODS

This study was conducted as a double-blind, parallel arm, individual, randomized controlled trial (RCT), registration number: ChiCTR2100042916. After obtaining the approval from the hospital ethics committee (No. K20-453, Date: 2020-12-31) and the patients’ written informed consents, we enrolled 218 elderly patients with lung cancer aged ≥70 years in our trial of a quantitative rapid pulmonary rehabilitation nursing program for patients undergoing the thoracic surgeries during 2021 (at the Outpatient Department of Thoracic Surgery; Shanghai Pulmonary Hospital, China). All the patients gave written informed consent. We allocated the patients to one of the following two groups:

### 1. Intervention arm – pulmonary rehabilitation nursing program.

### Description of intervention:

The intervention was delivered by the nurses employed in outpatient department of Thoracic surgery in Shanghai pulmonary hospital who are trained in pulmonary rehabilitation. The nurses gave out personalized breathing training and provided instructions about the exercises. Then, patients were made to perform the breathing exercises for approximately 30-minutes. The patients were also supervised by the nurses after the operation. The total duration of the intervention was three weeks.

### 2. Control arm – standard care (no intervention)

### Sample size calculation:

We used OpenEpi (v 3.01 updated on 2013, USA**)** to calculate the sample size on the basis of previous RCTs reporting a risk reduction in postoperative complications following pulmonary rehabilitation amongst patients with lung cancer (from 7.5% to 21%; 95% confidence interval (CI), 80% power, and a 5% alpha error). We estimated the minimum sample size to be 218 participants (109 in each group).

### Randomization details:

A statistician unrelated to this study used a block randomization technique with varying block sizes to generate a random sequence for 218 participants using the Random Allocation software. The statistician concealed the generated sequence using the SNOSE technique (sequentially numbered opaque serial envelopes). We assigned each eligible participant a unique number between one and 218 consecutively after the eligibility assessment; and, nurses in the department of pulmonary rehabilitation allocated each participant to either the intervention or control (standard care) arms. Blinding of the participants was not performed. However, blinding at the level of investigators and outcome assessors was done. Blinding at the investigator and outcome assessment level was possible as the intervention was delivered by the nurses who are not involved in the study. The same set of nurses were also trained in measuring the outcomes and they independently assessed the outcomes and entered the data in the anonymous format. The anonymized dataset was given to an independent statistician who has no knowledge about the allocation of intervention.

We obtained informed written consents and collected information regarding socio-demographic and baseline characteristics using a structured questionnaire. After admission and completion of an evaluation at the pulmonary rehabilitation nursing outpatient clinic, the pulmonary rehabilitation nurses gave out breathing trainers, instructed the patients in breathing training, and supervised the patients after the operation. The nurses collected data on variables such as the forced expiratory volume in one second (FEV_1_), the forced vital capacity (FVC), the FEV_1_/FVC% ratio, the maximum voluntary ventilation (MVV), the postoperative thoracic drainage volume, the postoperative hospital stay, the postoperative complication rate, and the postoperative quality of life during the baseline and end-line assessments.

The EQ-5D (EuroQOL five dimensions) comprises five questions on the following dimensions: mobility, self-care, activities (usual), pain and discomfort, and psychological status. These questions have three possible responses (one, indicating normal findings; two, indicating moderate problems, and three, indicating severe problems). We derived a summary index on the basis of the responses to these five dimensions after calculating a maximum score. The participants with the maximum score of one had the best health status (in contrast, higher scores indicated severe or frequent problems).

### Data Analysis:

We entered the study data into a Microsoft Excel software sheet and performed the analysis using the SPSS version 20 software (IBM Corp, Armonk, NY, USA). Continuous variables were expressed as means and standard deviations (SDs) or medians and interquartile ranges (IQRs) based on the normality of their distribution. We also applied unpaired T-tests to assess the statistical significance of difference in baseline continuous parameters and also post-intervention mean values between the two interventions. We summarized categorical variables as proportions. We tested for differences in baseline categorical parameters to assess statistical significance using a Chi-square test. Categorical outcome variables (postoperative complications) were reported as proportions with 95% confidence intervals (CIs) in both the intervention and control groups. Continuous parameters (FEV1, FVC, FEV1/FVC%, MVV) between the groups and over different time points were assessed using difference-in-difference analysis. In addition, we compared postoperative hospital stay and postoperative quality of life between the groups using an independent T-test; and, postoperative thoracic drainage volume using the Mann-Whitney test (the variable followed a non-normal distribution). The difference in postoperative complications was a categorical outcome and we assessed those using the Chi-square test. We considered all P values lower than 0.05 to be statistically significant.

## RESULTS

We screened 250 participants for eligibility criteria during the study period. Out of those, we enrolled 218 participants satisfying the eligibility criteria, with 109 participants in the intervention group and 109 participants in the control group. All the participants in the intervention and control groups were assessed for all the necessary variables during the follow-up (response rate 100%). We found similar baseline characteristics between the patients in the intervention and control groups. Table-I

**Table-I:** Baseline characteristics of participants in intervention & control arm (*n*=218).

Characteristics	Intervention arm	Control arm	p-value
Age (Mean ± SD)	73.1±0.31	73.8±0.31	0.13
***Gender***			
Male	54 (49.5)	68 (62.4)	0.06
Female	55 (50.5)	41 (37.6)	
***Smoking***			
Yes	34 (31.2)	34 (31.2)	1.00
No	75 (68.8)	75 (68.8)	
***Surgical Procedure***			
Lobectomy	67 (62.0)	69 (63.3)	0.71
Segmentectomy	32 (29.6)	34 (31.2)	
Wedge Resection	9 (8.4)	6 (5.5)	
***Pulmonary Function Test Parameters***			
Baseline FEV1 (Mean ± SD)	2.03 (0.04)	2.06 (0.04)	0.63
Baseline FEV1% (Mean ± SD)	95.2 (1.74)	93.2 (2.02)	0.44
Baseline FEV1/FVC (Mean ± SD)	104.7 (8.38)	95.5 (0.94)	0.27
Baseline MVV (Mean ± SD)	52.7 (1.88)	52.4 (1.92)	0.88
Baseline MVV% (Mean ± SD)	57.2 (1.85)	55.4 (2.00)	0.50

FEV-1, forced expiratory volume in one second; FVC, forced vital capacity; MVV, maximum voluntary ventilation.

Amongst the postoperative outcomes, first we assessed the quality of life of the study participants after completing the rehabilitation program. We found that the participants had improved their quality of life in all of the five dimensions under the EQ-5D (intervention arm mobility mean (SD), one (0) vs. control arm mobility mean (SD), 1.08 (0.28); *p*-value=0.002; intervention arm self-care mean (SD), 1.04 (0.19) vs. control arm self-care mean (SD), 1.18 (0.39); *p*-value <0.001; intervention arm activities mean (SD), 1.02 (0.13) vs. control arm activities mean (SD), 1.13 (0.34); *p*-value=0.002; intervention arm pain and discomfort mean (SD), one (0) vs. control arm pain and discomfort mean (SD), 1.11 (0.31); *p*-value<0.001; intervention arm anxiety mean (SD), 1.01 (0.13) vs. control arm anxiety mean (SD), 1.07 (0.26); *p*-value=0.05). Moreover, all the participants in the intervention group reported complete remission of their mobility and pain problems after the nursing pulmonary rehabilitation program. All the differences were statistically significant with a *p*-value lower than 0.05. The difference in the overall quality of life score was also found to be statistically significant between the intervention and control groups (intervention arm mean (SD), 0.85 (0.01) vs. control arm mean (SD), 0.84 (0.04); *p-*value=0.01).

The mean postoperative duration of hospital stay was shorter in the experimental group (7.18 days) than in the control group (7.61 days), but the difference was not statistically significant (p=0.33). We found similar mean tube indwelling times in both groups. More importantly, we found a significant difference in terms of the mean postoperative complications rate (3.7% in the experimental group vs. 11.9% in the control group) and this difference was statistically significant (p=0.02). Table-II

**Table-II:** Effect of pulmonary rehabilitation intervention on postoperative outcomes (quality of life, postoperative hospital stay and postoperative drainage volume and complications)

Characteristic	Intervention arm (Mean ± SD)	Control arm (Mean ± SD)	*p*-value
***Quality of Life (QOL)***			
EQ-5D-Mobility	1 (0)	1.08 (0.28)	0.002
EQ-5D-Self-care	1.04 (0.19)	1.18 (0.39)	<0.001*
EQ-5D-Activity	1.02 (0.13)	1.13 (0.34)	0.002*
EQ-5D-Pain & discomfort	1 (0)	1.11 (0.31)	<0.001*
EQ-5D-Anxiety	1.01 (0.13)	1.07 (0.26)	0.05*
Overall QOL	0.85 (0.01)	0.84 (0.04)	0.01*
***Other Postoperative outcomes***			
Length of hospital stay	7.18 (2.32)	7.61 (3.49)	0.33
Thoracic drainage (Median [IQR])^&^	130 (50-200)	150 (0-250)	0.38
Postoperative complications (n, %)^$^	4 (3.7)	13 (11.9)	0.02*

* *p*-value statistically significant,

& Mann-Whitney test,

$ Chi-square test.

We found all the pulmonary function variables to be similar between the intervention and control groups at baseline and post-intervention. The difference-in-difference, that is, the changes in pulmonary function parameters between the groups and over the different time points were also similar (p-value > 0.05). Table-III

**Table-III:** Difference-in-difference analysis to compare pulmonary function parameters between the intervention and control arms overtime (*n*=218)

Outcome variable	Pulmonary function test	t statistic	*p*-value
***FEV_1_***			
Baseline Mean difference	-0.03	0.47	0.64
Post-intervention Mean difference	0.002	0.02	0.99
Difference-in-difference	-0.03	0.34	0.73
***FEV_1%_***			
Baseline Mean difference	2.04	0.79	0.42
Post-intervention Mean difference	2.49	0.42	0.67
Difference-in-difference	-0.44	0.12	0.91
***FEV1/FVC%***			
Baseline Mean difference	9.27	1.46	0.14
Post-intervention Mean difference	16.55	1.14	0.25
Difference-in-difference	-7.28	0.77	0.44
***MVV***			
Baseline Mean difference	0.38	0.14	0.89
Post-intervention Mean difference	0.32	0.05	0.96
Difference-in-difference	0.06	0.01	0.99
***MVV%***			
Baseline Mean difference	1.81	0.69	0.49
Post-intervention Mean difference	5.69	0.94	0.35
Difference-in-difference	-3.88	0.98	0.33

## DISCUSSION

We tested a rapid and precise perioperative pulmonary rehabilitation nursing program performed before and/or after thoracic surgery in patients with lung cancer. Our program was comparatively shorter than the standard pulmonary rehabilitation program based on the standard ATS/ERS/BTS guidelines. We reduced the intervention duration preoperatively in line with the results of studies showing that a shorter duration was effective at improving the metabolism, lung mechanics, cardiovascular response, and muscle function of patients.^12^-^14^

The tested preoperative and postoperative nursing pulmonary rehabilitation program significantly reduced the postoperative complications rate and improved the quality of life of patients across all the variables in the EQ-5D. Other indicators also showed a better performance of the program in the experimental group, but we found similar mean values for FEV_1_, FVC, FEV_1_/FVC%, MVV, MVV%, length of hospital stay, and thoracic drainage volume.

Exercise tolerance has been a crucial component of the risk stratification before surgical resections amongst patients with lung cancer. Exercise interventions can improve the exercise capacity while reducing cardiopulmonary risks.^12^ Endurance training is essential during pulmonary rehabilitations. Supervised high-intensity interval training can be more effective than continuous training in healthy individuals. However, both methods have advantages that depend on the self-paced training program.^13^,^14^ Considering the functional capacity, the presence of significant co-morbidities, and the desaturation index of the patients during the exercises is important to design an optimal and personalized training program.^13^,^14^

Exercise capacity improvements, without significant changes in the pulmonary function variables of our patients are an unexpected finding in our study. However, this finding has been reported in other studies conducted among patients with COPD and other restrictive disorders.^15^,^16^

The role of nurses in the perioperative management of patients with lung cancer is often overlooked, but should be made an integral part of the postoperative management of patients with lung cancer, especially under the current expansion of integral nursing care in hospitals. Nurses may play an important role empowering patients and improving the postoperative management through a wide variety of care measures that include giving advice, educating patients and providing direct care. Our study findings show that our comprehensive nursing interventions on the basis of pulmonary rehabilitation measures are effective at preventing various complications that can occur following thoracic surgery. However, the evidence on the nursing pulmonary rehabilitation program improving the pulmonary function in patients with lung cancer remains unclear.

Our results showed a significant improvement in the quality of life of patients on the five tested dimensions of the EQ-5D. This proves that our nursing pulmonary rehabilitation program has a wholesome effect on the patients’ postoperative outcomes positively affecting their physical, social and mental health. However, previous studies found no significant improvement in the quality of life postoperatively following a pulmonary rehabilitation program^17^-^21^ Some researchers have suggested that preoperative rehabilitation programs may not result in a significant change in the quality of life of patients due to a possible ceiling effect.^17^ Indeed, trials exploring the effectiveness of such a program amongst patients with lung cancer reported such findings.^18^-^21^ We cannot make conclusions about the quality of life on the basis of our results given the differences in the tools, intervention designs, and/or extent of the surgical operations in the different relevant studies. We used the EQ-5D, which is a worldwide standard tool for measuring quality of life; and, the questionnaire has been validated for use and a valuation set for our country is available. Thus, given the conflicting results, comparing ours to those in future studies using similar measurement tools with good validity and reliability is essential.^22^,^23^

### Strength of the study:

We conducted a randomized controlled trial (the highest form of evidence to assess the effectiveness of an intervention). We comprehensively assessed a wide range of outcomes postoperatively. We did not have any patients lost to follow-up during the study. In addition, we used the standard EQ-5D tool to assess the quality of life of the patients postoperatively. The lack of a significant effect of the post-surgical pulmonary rehabilitation interventions on the recovery and preservation of pulmonary function in elderly patients, may reflect a non-significant association between the postoperative pulmonary rehabilitation training and the patient’s lung function preservation. However, given that elderly patients usually present underlying diseases and that the occurrence of complications usually leads to serious adverse events and even perioperative death, our results are important because they show that the patients in the pulmonary rehabilitation intervention group had a lower incidence of complications than those in the control group. Thus, our findings suggest that our pulmonary rehabilitation intervention successfully helped the overall rehabilitation of elderly patients and integrate the intervention in primary healthcare settings.^24^ We cannot show similar effects for the perioperative mortality or long-term survival between the two groups, but our intervention seemed to promote the physical recovery and quality of life of our elderly patients.

### Limitations

Blinding of the participants could not be done due to the nature of the intervention; however, the investigator and outcome assessor were blinded to the intervention. We failed to evaluate the mental and emotional aspects of the quality of life of our patients and focused instead on physical aspects.

## CONCLUSION

Our results showed a significant improvement in the quality of life of patients on the five tested dimensions of the EQ-5D. However, we found a non-significant effect of the post-surgical pulmonary rehabilitation interventions on the recovery and preservation of pulmonary function in elderly patients. Nonetheless, strengthening pulmonary rehabilitation nursing interventions for elderly patients with lung cancer during the perioperative period, which can possibly reduce their postoperative complications and promote their rapid recovery.

### Authors’ contributions:

**BC** conceived and designed the study.

**XY, XW and YM** collected the data and performed the analysis.

**BC** was involved in the writing of the manuscript and is responsible for the integrity of the study.

All authors have read and approved the final manuscript.

## References

[1] Siddiqui F, Vaqar S, Siddiqui AH (2022). Lung Cancer.

[2] Venuta F, Diso D, Onorati I, Anile M, Mantovani S, Rendina EA (2016). Lung cancer in elderly patients. J Thorac Dis.

[3] Levett DZH, Grocott MPW (2015). Cardiopulmonary exercise testing, prehabilitation, and Enhanced Recovery After Surgery (ERAS). Can J Anaesth.

[4] Brunelli A, Charloux A, Bolliger CT, Rocco G, Sculier JP, Varela G (2009). The European Respiratory Society and European Society of Thoracic Surgeons clinical guidelines for evaluating fitness for radical treatment (surgery and chemoradiotherapy) in patients with lung cancer. Eur J Cardio-Thorac Surg.

[5] Hou R, Miao F, Jin D, Duan Q, Yin C, Feng Q, Wang T (2022). General Anesthesia for Patients with Chronic Obstructive Pulmonary Disease and Postoperative Respiratory Failure: A Retrospective
Analysis of 120 Patients. Front Physiol.

[6] Tsubochi H, Shibano T, Endo S (2018). Recommendations for perioperative management of lung cancer patients with comorbidities. Gen Thorac Cardiovasc Surg.

[7] Huang WW, Zhu WZ, Mu DL, Ji XQ, Nie XL, Li XY (2018). Perioperative Management May Improve Long-term Survival in Patients After Lung Cancer Surgery: A Retrospective Cohort Study. Anesth Analg.

[8] Zhang F, Zhong Y, Qin Z, Li X, Wang W (2021). Effect of muscle training on dyspnea in patients with chronic obstructive pulmonary disease: A meta-analysis of randomized controlled trials. Medicine.

[9] Asher A, Myers JS (2015). The effect of cancer treatment on cognitive function. Clin Adv Hematol Oncol HO.

[10] Wang H, Liu X, Rice SJ, Belani CP (2016). Pulmonary Rehabilitation in Lung Cancer. PM R.

[11] Corhay JL, Dang DN, Van Cauwenberge H, Louis R (2014). Pulmonary rehabilitation and COPD: providing patients a good environment for optimizing therapy. Int J Chron Obstruct Pulmon Dis.

[12] Hoogeboom TJ, Dronkers JJ, Hulzebos EHJ, van Meeteren NLU (2014). Merits of exercise therapy before and after major surgery. Curr Opin Anaesthesiol.

[13] Vagvolgyi A, Rozgonyi Z, Kerti M, Vadasz P, Varga J (2017). Effectiveness of perioperative pulmonary rehabilitation in thoracic surgery. J Thorac Dis.

[14] Gao M, Huang Y, Wang Q, Liu K, Sun G (2022). Effects of High-Intensity Interval Training on Pulmonary Function and Exercise Capacity in Individuals with Chronic Obstructive Pulmonary Disease: A Meta-Analysis and Systematic Review. Adv Ther.

[15] Haave E, Hyland ME, Engvik H (2007). Improvements in exercise capacity during a 4-weeks pulmonary rehabilitation program for COPD patients do not correspond with improvements in self-reported health status or quality of life. Int J Chron Obstruct Pulmon Dis.

[16] Arnold MT, Dolezal BA, Cooper CB (2020). Pulmonary Rehabilitation for Chronic Obstructive Pulmonary Disease: Highly Effective but Often Overlooked. Tuberc Respir Dis.

[17] Almeida P, Rodrigues F (2014). Exercise training modalities and strategies to improve exercise performance in patients with respiratory disease. Rev Port Pneumol.

[18] Cui L, Liu H, Sun L (2019). Multidisciplinary respiratory rehabilitation in combination with non-invasive positive pressure ventilation in the treatment of elderly patients with severe chronic obstructive pulmonary disease. Pak J Med Sci.

[19] Wang L, Yu M, Ma Y, Tian R, Wang X (2022). Effect of Pulmonary Rehabilitation on Postoperative Clinical Status in Patients with Lung Cancer and Chronic Obstructive Pulmonary Disease: A Systematic Review and Meta-Analysis. Evid Based Complement Alternat Med.

[20] Yu Z, Xie G, Qin C, Qiu X (2022). Study on the Benefit of Postoperative Exercise Rehabilitation in Patients with ?Lung Cancer Complicated with Chronic Obstructive Pulmonary Disease. Zhongguo Fei Ai Za Zhi.

[21] Alyami RM, Alhowikan AM, Alharbi AR, Al-Nafisah G (2020). Impact of supervised exercise training on pulmonary function parameters, exercise capacity and Irisin Biomarker in Interstitial lung disease patients. Pak J Med Sci.

[22] Cavalheri V, Burtin C, Formico VR, Nonoyama ML, Jenkins S, Spruit MA (2019). Exercise training undertaken by people within 12. amonths of lung resection for non-small cell lung cancer. Cochrane Database Syst Rev.

[23] Ann-Yi S, Bruera E (2022). Psychological Aspects of Care in Cancer Patients in the Last Weeks/Days of Life. Cancer Res Treat.

[24] Saeed S, Siddiqui M, Altaf R (2022). The Obstructive Lung Diseases Program: Integrated obstructive lung disease services within primary care in Pakistan. Pak J Med Sci.

